# Estrogen receptor β upregulated by lncRNA-*H19* to promote cancer stem-like properties in papillary thyroid carcinoma

**DOI:** 10.1038/s41419-018-1077-9

**Published:** 2018-11-02

**Authors:** Mei Li, Hui-Fang Chai, Fei Peng, Yu-Ting Meng, Li-Zhi Zhang, Lin Zhang, Hong Zou, Qi-Lan Liang, Man-Man Li, Kai-Ge Mao, Dong-Xu Sun, Meng-Ying Tong, Zi-Qian Deng, Zhi-Jie Hou, Yi Zhao, Jia Li, Xiao-Chao Wang, Sha-Sha Lv, Qing-Qing Zhang, Xiao Yu, Eric W.-F. Lam, Quentin Liu, Xiao-Nan Cui, Jie Xu

**Affiliations:** 1 The First Affiliated Hospital, Institute of Cancer Stem Cell, Dalian Medical University, Dalian, China; 2 State Key Laboratory of Oncology in South China, Cancer Center, Sun Yat-sen University, Guangzhou, China; 3 Dongfang Hospital, Key Laboratory of Health Cultivation of the Ministry of Education, Beijing University of Chinese Medicine, Beijing, China; 4 Department of Pathology, The First Affiliated Hospital of Dalian Medical University, Dalian, China; 5 Dalian Municipal Women And Children’s Medical Center, Dalian, China; 6 Departmemt of Pathology, Dalian Medical University, Dalian, China; 7 Department of Surgery and Cancer, Imperial College London, London, W12 0NN UK

## Abstract

Estrogen receptor β (ERβ) plays critical roles in thyroid cancer progression. However, its role in thyroid cancer stem cell maintenance remains elusive. Here, we report that ERβ is overexpressed in papillary thyroid cancer stem cells (PTCSCs), whereas ablation of ERβ decreases stemness-related factors expression, diminishes ALDH^+^ cell populations, and suppresses sphere formation ability and tumor growth. Screening estrogen-responsive lncRNAs in PTC spheroid cells, we find that lncRNA-*H19* is highly expressed in PTCSCs and PTC tissue specimens, which is correlated with poor overall survival. Mechanistically, estradiol (E2) significantly promotes *H19* transcription via ERβ and elevates *H19* expression. Silencing of *H19* inhibits E2-induced sphere formation ability. Furthermore, *H19* acting as a competitive endogenous RNA sequesters miRNA-3126-5p to reciprocally release ERβ expression. ERβ depletion reverses *H19*-induced stem-like properties upon E2 treatment. Appropriately, ERβ is upregulated in PTC tissue specimens. Notably, aspirin attenuates E2-induced cancer stem-like traits through decreasing both *H19* and ERβ expression. Collectively, our findings reveal that ERβ-*H19* positive feedback loop has a compelling role in PTCSC maintenance under E2 treatment and provides a potential therapeutic targeting strategy for PTC.

## Introduction

Papillary thyroid carcinoma (PTC) is one of the most common thyroid neoplasms, which exhibits multicentricity in the thyroid gland and frequently metastasizes to the regional lymph nodes, thereby increasing both morbidity and mortality^1^. Increasing evidence indicates that papillary thyroid cancer stem cells (PTCSCs) play an important role in the progression of PTC^2^. For example, stem cell marker *POU5F1* is highly expressed in CD44^+^/CD24^−^ subpopulation and tumorigenic thyrospheroid cells from PTC^3^. Tumor spheroids from PTC samples are more resistant to chemotherapeutics, including bortezomib, taxol, cisplatin, etoposide, doxorubicin, and vincristine, than non-spheroid PTC cells^4^. In PTC tissues, a positive correlation has been found between stemness-related gene expression and tumor, lymph node, metastasis (TNM) staging^5^. E2 is the most potent estrogen, which has a high affinity to estrogen receptor α (ERα), estrogen receptor β (ERβ), and Peroxisome proliferator-activated receptor gamma (PPAR-γ or PPARg)^6,7^. E2 enhances migration and invasion of PTC cells modulated by E-cadherin, vimentin and MMP-9^8^. Moreover, E2 stimulation elevates stemness-related gene expression in PTC cells and promotes motility and tumorigenicity of PTCSCs in vivo^9^. However, the molecular mechanism of estrogen regulating PTCSC maintenance remains poorly understood.

Long noncoding RNAs (lncRNAs) are a class of transcripts longer than 200 nucleotides but with no protein-coding potential, which play a crucial role in regulating cancer cell stemness. For example, recent studies show that knockdown of *NEAT1* inhibits glioma stem cells progression via let-7e-NRAS axis^10^. LncRNA *H19* increases core pluripotency factor LIN28 expression by blocking the bioactivity of let-7 to promote breast cancer stem cell maintenance^11^. LncRNA-*DILC* also attenuates liver cancer stem cell expansion through inhibiting the autocrine of IL6/STAT3 signaling^12^. In addition, *ElncRNA1* is transcriptionally regulated by E2 through ERα-estrogen response element pathway to promote epithelial ovarian cancer cell proliferation^13^. Furthermore, E2 treatment also drives Sp1 to increase lncRNA *MALAT1* expression and epigenetically controls various physiological processes of osteosarcoma cells^14^. Although accumulating studies have indicated lncRNAs play important roles in maintaining CSCs and could be regulated by estrogen signaling in diverse cancers, little is known about the mechanism by which lncRNAs modulate E2-induced PTCSCs.

Emerged evidence has suggested that estrogen receptors (ERs) play pivotal roles in the pathogenesis of PTC. For example, ERα can trigger autophagy via activating ROS and ERK1/2 pathways to promote cell proliferation and inhibit apoptosis in PTC cells^15^. ERβ is associated with apoptosis and growth inhibition, providing a negative correlation with mutant p53 in female PTC patients of reproductive age^16^. Moreover, reciprocal interactions between ERβ and PPARg significantly inhibit PTC cell proliferation and migration, while ERα offsets the inhibitory effect of PPARg on cellular functions^17^. In addition, ER-elevated OCT4 expression promotes self-renewal of the human breast cancer stem cells^18^. Furthermore, thyroid stem and progenitor cells derived from nodular goiters express higher levels of ERα and ERβ compared with the differentiated thyrocytes^19^. However, the underlying molecular mechanism whereby ER promotes PTC stemness is again still unclear.

Here, we demonstrate that ERβ is enriched in PTCSCs and contributes to PTCSC maintenance. Meanwhile, lncRNA *H19* is highly expressed in PTCSCs and PTC tissue specimens. E2 promotes *H19* transcription via ERβ. Ablation of *H19* antagonizes E2-induced cancer stem-like properties in PTC cells. Moreover, ERβ is elevated through *H19*/miR-3126-5p signaling axis. ERβ depletion markedly reverses *H19*-mediated PTC stem-like capability under E2 treatment. ERβ is also upregulated in PTC tissue specimens. Importantly, aspirin suppresses E2-induced cancer stem-like characteristics through decreasing both ERβ and *H19* expression. Taken together, our study identifies a novel mechanism of E2-induced ERβ-*H19* positive regulatory circuit in PTCSC maintenance, providing a potential therapeutic strategy for PTC.

## Results

### ERβ contributes to PTCSCs

**Fig. 1:**
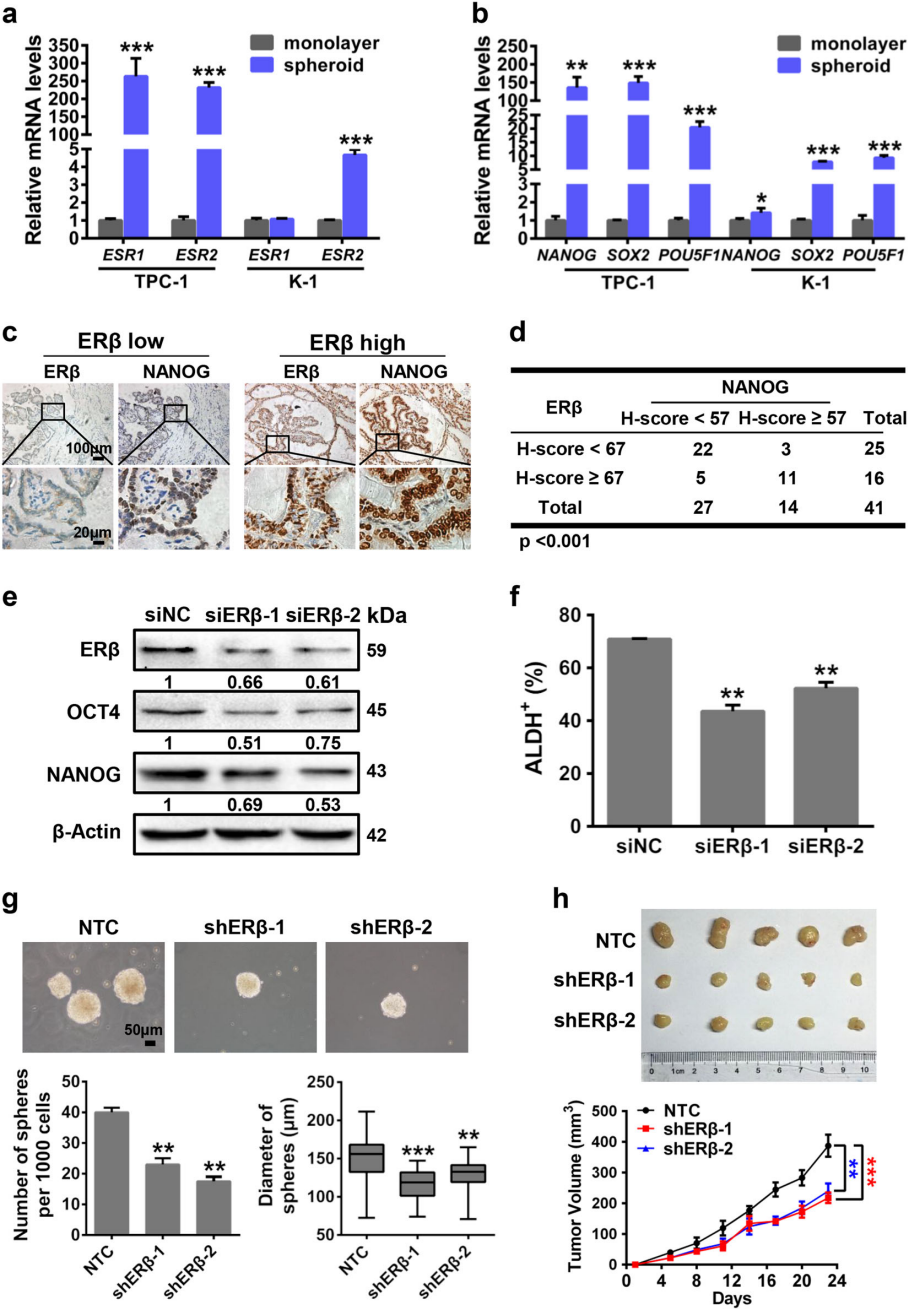
ERβ contributes to papillary thyroid CSCs. **a** *ESR1* and *ESR2* mRNA expression in the spheroid cells and monolayer cells of TPC-1 cells and K-1 cells were analyzed by RT-qPCR. Data were shown as means ± SD (*n* = 3, ***P < 0.001). **b** A panel of core pluripotency factors (*NANOG*, *SOX2,* and *POU5F1*) were measured between spheroid cells and monolayer cells from both TPC-1 cells and K-1 cells. Data were shown as means ± SD (*n* = 3, *P < 0.05, **P < 0.01 and ***P < 0.001). **c** PTC tissue specimens were subjected to IHC staining by ERβ and NANOG antibodies. Representative images were presented. Scale bar, 100 or 20 μm. **d** The correlation between the ERβ and NANOG expression in PTC tissues from 41 patients. ***P < 0.001. **e** The protein levels of ERβ and stemness-related factors NANOG and OCT4 were measured in siNC and siERβ K-1 cells. β-Actin acted as the loading control. **f** The proportions of ALDH^+^ cells were compared between siNC and siERβ K-1 cells. **g** Sphere formation ability was performed in ERβ depletion K-1 cells. Representative images were presented (up), the scale bar represents 50 μm. The numbers and size of spheres were counted after culture for 10 days (down). Data were shown as means ± SD (*n* = 3, **P < 0.01 and ***P < 0.001). **h** BALB/c nude male mice (*n* = 5) were subcutaneously inoculated with equal number of single cells (1 × 10^6^ cells). The tumors were harvested at day 23 after injection (upper panel). Tumor volumes were monitored as described (lower panel)

As the effect of estrogen is predominantly mediated through ERα and ERβ, we first examined whether ERα and ERβ are involved in PTC stemness. To this end, we performed sphere formation assay to enrich PTCSCs. The mRNA levels of *ESR1* and *ESR2* were compared between spheroid and monolayer cells. The results showed that *ESR2* mRNA expression was remarkably elevated in both TPC-1 spheroid cells and K-1 spheroid cells compared to their monolayer counterparts (Fig. 1a). Spheroid cells exhibited much higher mRNA expression of stemness-related factors, including *NANOG*, *SOX2*, and *POU5F1*, in both TPC-1 and K-1 cells (Fig. 1b). Conversely, the spheroid cells exhibited a relative reduction of thyroid differentiation markers including Thyroglobulin (*Tg*) and thyroid stimulating receptor (TSHR) in both TPC-1 and K-1 cells (Supplementary Figure 1a). Next, we measured ERβ and stemness-related protein NANOG in the same paraffin-embedded tissue specimens and found that ERβ was positively correlated with NANOG expression in the PTC tissues (Fig. 1c, d). Furthermore, ablation of ERβ by siRNAs significantly decreased NANOG and OCT4 protein expression in both K-1 cells (Fig. 1e) and TPC-1 cells (Supplementary Figure 1b). ERβ knockdown diminished ALDH^+^ cell populations in both K-1 cells (Fig. 1f) and TPC-1 cells (Supplementary Figure 1c). In addition, ERβ was knocked down by shRNA in both K-1 (Supplementary Figure 1d) and TPC-1 cells (Supplementary Figure 1e). Sphere formation assay showed that ERβ depletion significantly decreased spheroid numbers and diameters in both K-1 (Fig. 1g) and TPC-1 (Supplementary Figure 1f) cells. Moreover, the effect of ERβ on tumorigenesis was further examined in nude mice by injecting with control K-1 cells (NTC; non-targeting control), shERβ-1 K-1 cells (shERβ-1) and shERβ-2 K-1 cells (shERβ-2). As shown in Fig. 1h, the tumor volumes in shERβ-1 and shERβ-2 groups were apparently smaller than NTC group, indicating that ERβ is critical for PTCSC maintenance.

### *H19* is highly expressed in PTCSCs and PTC tissue specimens

**Fig. 2:**
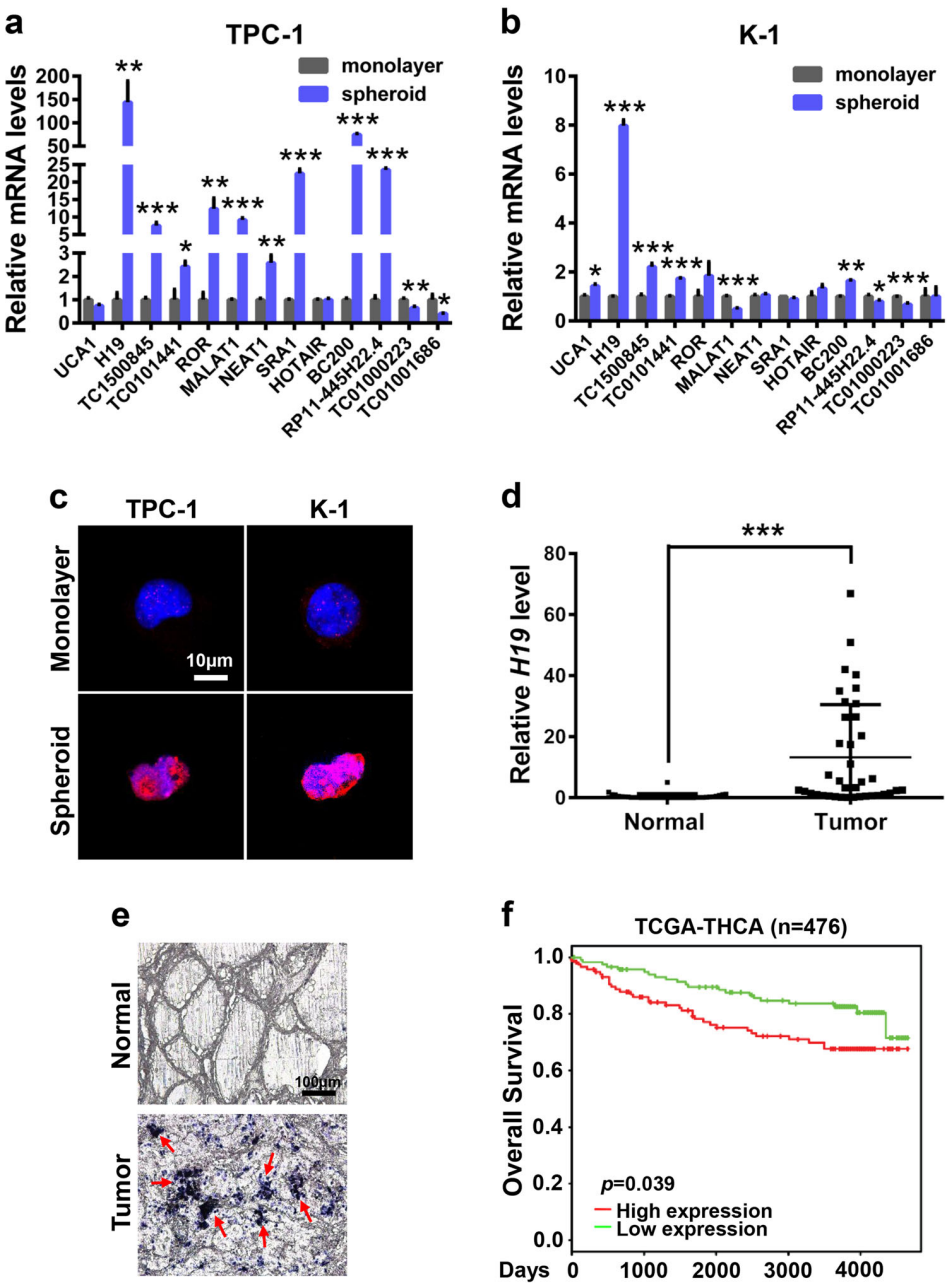
*H19* expression is elevated in PTCSCs and PTC tissue specimens. **a** RT-qPCR analysis of the indicated lncRNA levels in the TPC-1 spheroid cells and TPC-1 monolayer cells. Data were shown as means ± SD (*n* = 3) *P < 0.05, **P < 0.01 and ***P < 0.001. **b** RT-qPCR analysis of the indicated lncRNA levels in the K-1 spheroid cells and K-1 monolayer cells. Data were shown as means ± SD (*n* = 3) *P < 0.05, **P < 0.01 and ***P < 0.001. **c** The in situ expression of *H19* RNA was detected by FISH assay. The red fluorescent represents *H19* RNA probe, and the blue fluorescent signal represents nuclear DNA counterstained with DAPI. The scale bar represents 10 μm. **d** *H19* expression in PTC tissues and adjacent normal tissues were analyzed by RT-qPCR assay (*n* = 38). The relative *H19* level was normalized to *ACTB*. The statistical difference was analyzed using the paired *t*-test. ***P < 0.001. **e** In situ analysis with a DIG-labeled *H19* probe in PTC tissue specimens and adjacent normal tissue specimens. The scale bar represents 100 μm. **f** Kaplan–Meier overall survival plots of 476 thyroid cancer patients created using PROGgeneV2, data set from TCGA-THCA. Patients were classified into *H19*-high and *H19*-low subgroups and analyzed as indicated

The fact that lncRNAs can be induced by estrogen and are widely involved in cancer stem cell maintenance prompted us to investigate whether lncRNA plays a critical role in regulating PTCSCs. To this end, we compared the expression levels of 13 potential estrogen-responsive lncRNAs (*UCA1*, *H19*, *TC1500845*, *TC0101441*, *ROR*, *MALAT1*, *NEAT1*, *SRA1*, *HOTAIR*, *BC200*, *RP11-445H22.4*, *TC01000223* and *TC01001686*) between spheroids and monolayer cells^13,14,20–27^. The results showed that *H19* was the highest expressing lncRNA in spheroids as well as the highest differentially expressed lncRNA when compared with monolayer cells (Fig. 2a, b). Consistently, *H19* expression was much higher in spheroid cells than in monolayer cells through FISH assay (Fig. 2c). Moreover, *H19* expression level was significantly higher in PTC tissue specimens compared with the adjacent tissue specimens (Fig. 2d, e). Furthermore, the Kaplan–Meier survival analysis demonstrated that high *H19* levels were a strong indicator for poor overall survival of thyroid cancers in TCGA database (Fig. 2f), suggesting a remarkably unfavorable prognosis and shorter lifespan. In summary, *H19* is highly expressed in PTCSCs and PTC tissue specimens.

### *H19* depletion reverses E2-induced stem-like properties in PTC cells

**Fig. 3:**
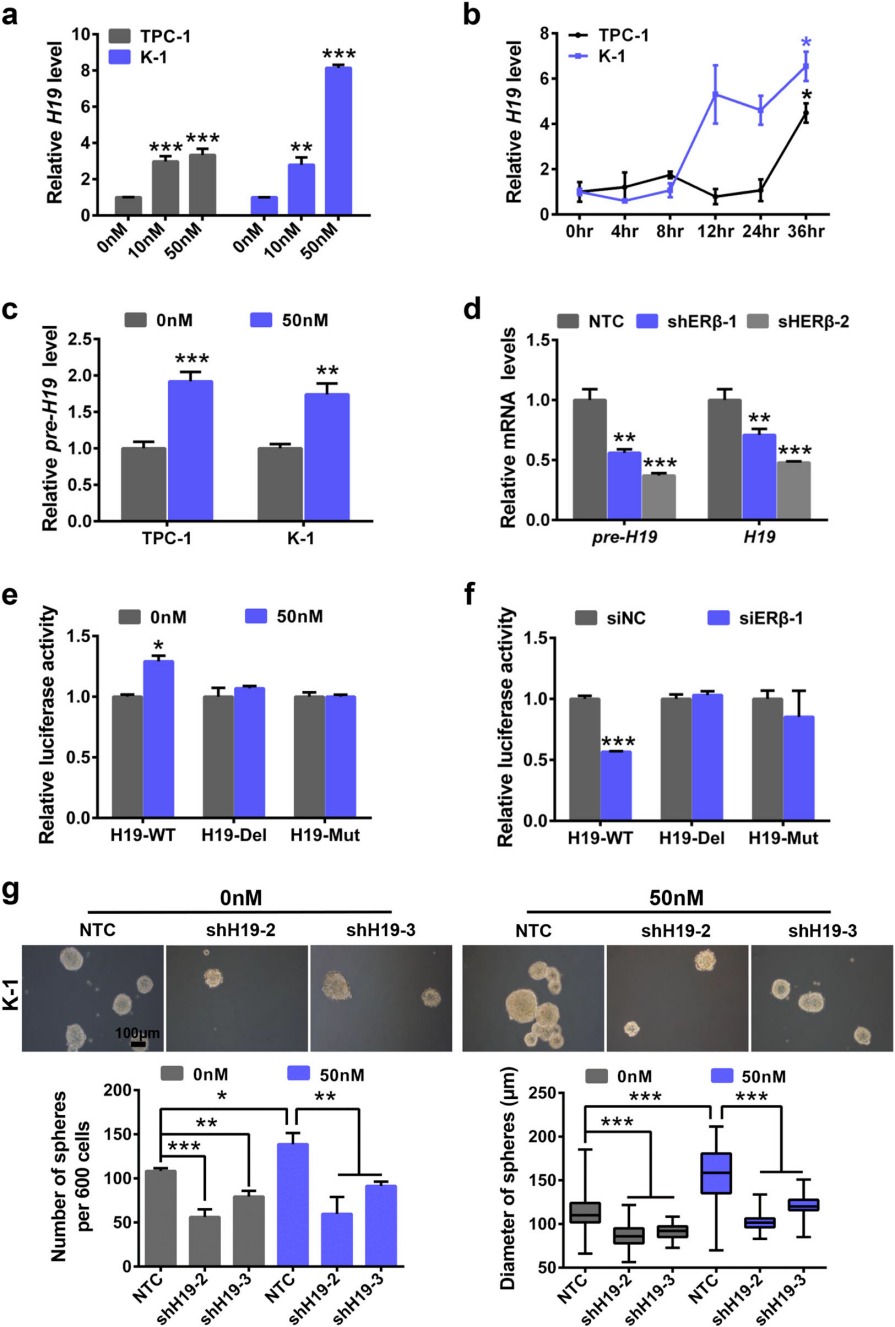
*H19* mediates E2-induced stem-like properties in PTC cells. **a** TPC-1 and K-1 cells were treated with various concentrations (0, 10, and 50 nM) of E2 for 36 h. Total RNA was extracted and subjected to detect *H19* expression by RT-qPCR analysis. The relative *H19* level was normalized to *ACTB*. Data were shown as means ± SD. (*n* = 3, **P < 0.01 and ***P < 0.001). **b** TPC-1 and K-1 cells were treated with 50 nM E2 for 0, 4, 8, 12, 24, 36 h. Total RNA was extracted and subjected to detect *H19* expression by RT-qPCR analysis. The relative *H19* level was normalized to *ACTB*. Data were shown as means ± SD. (*n* = 3, *P < 0.05). **c** TPC-1 and K-1 cells were treated with E2 (50 nM) for 36 h. Total RNA was extracted and subjected to detect pre-*H19* expression by RT-qPCR. The relative pre-*H19* levels were normalized to *ACTB*. Data were shown as means ± SD. (*n* = 3, **P < 0.01 and ***P < 0.001). **d** Total RNA was extracted in NTC, shERβ-1, shERβ-2 K-1 cells. Pre-*H19* and *H19* were detected by RT-qPCR assay. The relative RNA levels were normalized to *ACTB*. Data were shown as means ± SD (*n* = 3, **P < 0.01 and ***P < 0.001). **e** pGL3-EV, pGL3-H19-WT, -H19-Del, or -H19-Mut along with pRL-SV40 were transfected into K-1 cells with E2 treatment. After 24 h, dual-luciferase reporter assays were performed (*n* = 3, *P < 0.05). **f** siNC and siERβ were co-transfected into K-1 cells with pGL3-EV, pGL3-H19-WT, -H19-Del, or -H19-Mut along with pRL-SV40. After 24 h, dual-luciferase reporter assays were performed (*n* = 3, ***P < 0.001). **g** Sphere formation abilities of K-1 under different conditions were compared. Representative images were presented, the scale bar represents 100 μm. The numbers and size of spheres were counted after culture for 10 days. Data were shown as means ± SD. (*n* = 3, *P < 0.05, **P < 0.01 and ***P < 0.001)

To further explore whether *H19* is involved in E2-induced PTCSC maintenance, we performed sphere formation assay upon treatment with E2 in PTC cells. Both sphere numbers and diameters were markedly elevated upon E2 treatment in both TPC-1 cells and K-1 cells (Supplementary Figure 2a and b). In addition, we conducted RT-qPCR assay to further explore the effects of E2 on PTC stemness. E2 increased substantially the mRNA expression levels of stemness-related factors, including *NANOG*, *SOX2* and *POU5F1*, in both TPC-1 cells and K-1 cells (Supplementary Figure 2c). PTC cells were also treated with various doses (0, 10, and 50 nM) of E2 for 36 h. As shown in Fig. 3a, E2 significantly increased *H19* RNA levels of PTC cells in a dose-dependent manner. Consistently, *H19* RNA expression was also elevated by E2 treatment (50 nM) in a time-dependent manner in both TPC-1 cells and K-1 cells (Fig. 3b). Furthermore, E2 promoted *H19* pre-RNA expression (Fig. 3c) and increased *ESR2* but not *ESR1* mRNA expression (Supplementary Figure 2d) in both TPC-1 and K-1 cells, which prompted that E2 regulates *H19* transcription through ERβ. Indeed, silencing of ERβ significantly decreased both pre-*H19* and *H19* RNA levels (Fig. 3d). To determine transcription activity of *H19*, *H19* promoter sequence (H19-WT) and the *H19* promoter sequence with truncated ERE segment (H19-Del) and the *H19* promoter with ERE domain (H19-Mut) were cloned into the pGL3 vector (Supplementary Figure 2e), respectively. E2 treatment promoted H19-WT luciferase activity, while it had no effects on H19-Del and H19-Mut activities (Fig. 3e). Conversely, depletion of ERβ dramatically attenuated H19-WT luciferase activity, whereas it caused no changes in H19-Del and H19-Mut activities (Supplementary Figure 2f and Fig. 3f). These data show that E2 promotes stem-like traits and increases *H19* transcription in PTC cells.

We next investigated whether *H19* mediated E2-derived cancer stem-like traits in PTC cells. To achieve this, *H19* was knocked down by shRNA in TPC-1 cells and K-1 cells (Supplementary Figure 2g). Next, we performed sphere formation assay and observed that E2 treatment increased spheroid numbers and diameters, while *H19* knockdown attenuated sphere formation capacity. Depletion of *H19* significantly reversed E2-induced sphere formation capability in both K-1 cells (Fig. 3g) and TPC-1 cells (Supplementary Figure 2h). These data provide evidence to suggest that *H19* plays an essential role in promoting cancer stem-like characteristics induced by E2 in PTC cells.

### ERβ regulated by *H19*/miR3126-5p signaling axis promotes cancer stem-like properties upon E2 treatment

**Fig. 4:**
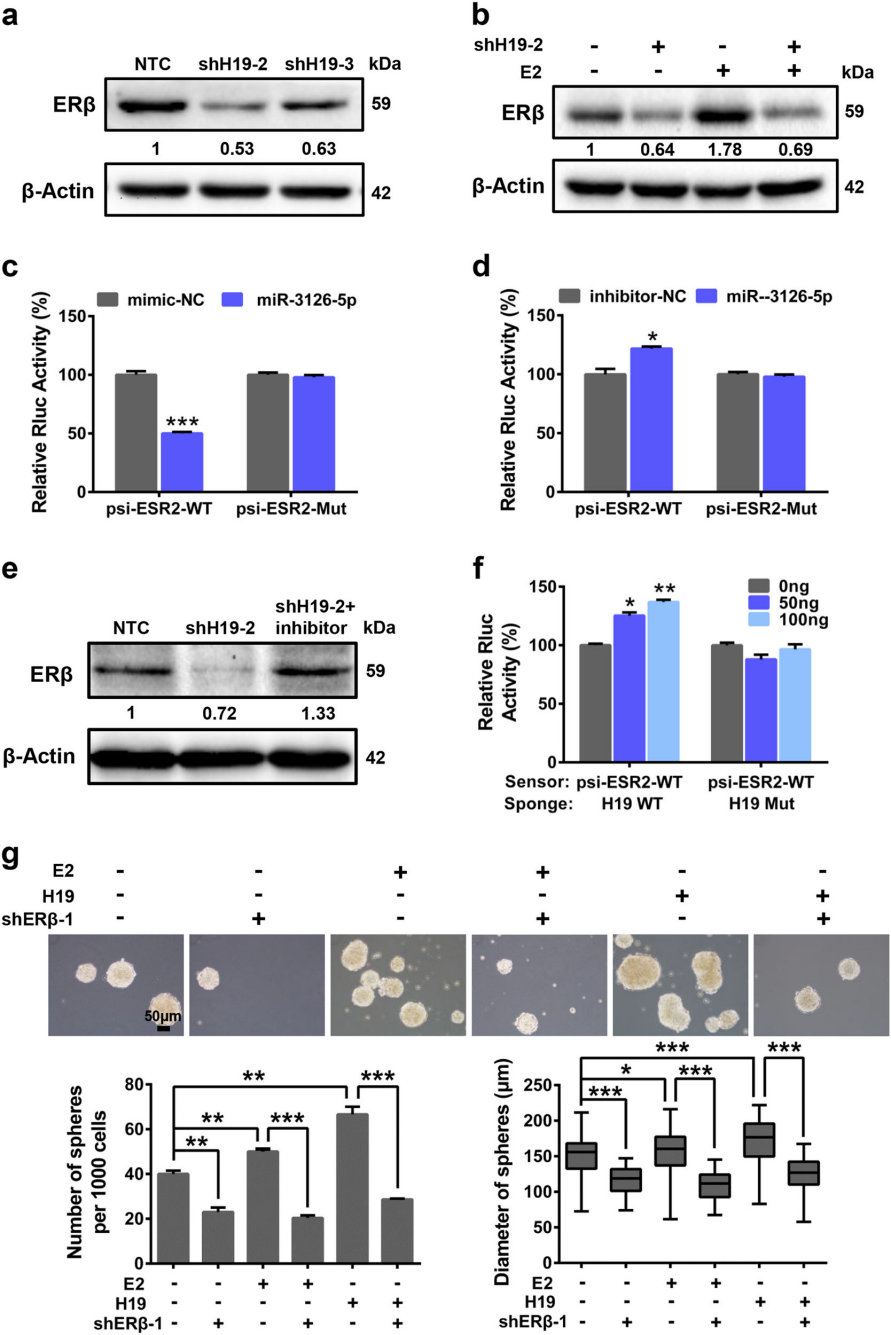
*H19*/miR-3126-5p/ERβ regulated stem-like properties under E2 treatment in PTC cells. **a** ERβ expression was analyzed by western blotting in K-1 shH19 cells and NTC cells. β-Actin acted as the loading control. **b** K-1 (shH19-2) cells and NTC cells were treated with or without E2 for 36 h. ERβ expression was analyzed by western blotting. β-Actin acted as the loading control. **c** The psi-ESR2-WT or -Mut, and miR-3126-5p mimic along with negative control (NC) were co-transfected into K-1 cells. The regulation of *ESR2* by miR-3126-5p was measured by luciferase assay (*n* = 3, ***P < 0.001). **d** The psi-ESR2-WT or -Mut, and miR-3126-5p inhibitor in parallel with negative control (NC) were co-transfected into K-1 cells. The regulation of *ESR2* by miR-3126-5p was measured by luciferase assay (*n* = 3, *P < 0.05). **e** ERβ expression was examined in shH19 cells and miR-3126-5p inhibitor transfected shH19 cells compared with NTC K-1 cells. β-Actin was used as the loading control. **f** K-1 cells were transfected with miR-3126-5p sensor (psi-ESR2-WT) together with 0, 50, and 100 ng of wild-type H19 (WT) or mutant H19 (Mut) plasmids, and dual-luciferase reporter activity was analyzed (*n* = 3, *P < 0.05, **P < 0.01). **g** Sphere formation abilities of shERβ K-1 cells and NTC cells under different conditions were compared. Representative images were presented, counted at 20×, the scale bar represents 50 μm. The numbers and size of spheres were counted after culture for 10 days. Data were shown as means ± SD. (*n* = 3, *P < 0.05, **P < 0.01 and ***P < 0.001)

We next investigated the molecular mechanism whereby ERβ regulates *H19*-induced stem-like properties upon E2 treatment. We firstly measured the expression of *ESR2* in the *H19*-knockdown (shH19) PTC cells. The result showed that there were no significant changes in *ESR2* mRNA levels in the PTC cells upon *H19* knockdown (Supplementary Figure 3a), while *H19* depletion decreased ERβ protein levels in K-1 cells (Fig. 4a). E2 treatment increased ERβ expression, which could be attenuated by silencing *H19* in K-1 cells (Fig. 4b and Supplementary Figure 3b). To confirm whether *H19* acting as a competitive endogenous sponge interacts with miRNAs to release ERβ expression, we searched for miRNAs that interact with *H19* and also target 3′UTR region of *ESR2* by bioinformatic tools. The mimics of six identified miRNAs (Supplementary Figure 3c), including miR-4268, miR-3198, miR-876-3p, miR-1976, miR-3126-5p and miR-127-5p, were transfected into K-1 cells. The results showed that the miR-3126-5p mimetic significantly decreased ERβ protein expression (Supplementary Figure 3d), while miR-3126-5p inhibitor remarkably increased ERβ protein level (Supplementary Figure 3e). Moreover, wild-type *ESR2* 3′UTR sequence including the putative miRNA-3126-5p response element (MRE) and the MRE mutant were cloned into the psiCHECK2 vector to give rise to psi-ESR2-WT and psi-ESR2-Mut (Supplementary Figure 3f), respectively. The psi-ESR2-WT and psi-ESR2-Mut vectors were then independently transfected into K-1 cells together with miR-3126-5p mimic or inhibitor in parallel with negative controls. The results showed that miR-3126-5p mimic repressed, but miR-3126-5p inhibitor increased, the relative luciferase activity of reporter psi-ESR2-WT, whereas both of them had no effects on psi-ESR2-Mut (Fig. 4c, d). Consistently, miR-3126-5p released by shH19 decreased ERβ expression, which could be rescued by the miR-3126-5p inhibitor in K-1 cells (Fig. 4e). Furthermore, we found that in K-1 cells the relative luciferase activity of psi-ESR2-WT (sensor) was induced by increasing amounts of wide-type *H19* (H19-WT, sponge of miR-3126-5p), but not by *H19* with the miR-3126-5p binding sites mutated (H19-Mut) in a dose-dependent manner (Fig. 4f).

Next, *H19*-overexpressing plasmid was transiently transfected into shERβ or non-targeting control (NTC) transduced K-1 cells (Supplementary Figure 3g). Sphere formation assay showed that E2 treatment or *H19* overexpression significantly promoted sphere formation capacities, whereas depletion of ERβ restricted *E2*- or *H19*-induced stem-like properties in K-1 cells (Fig. 4g). These results support the idea that *H19*/miR3125-5p regulates stem-like properties upon E2 treatment through ERβ in PTC cells.

### ERβ is upregulated in PTC tissue specimens

**Fig. 5:**
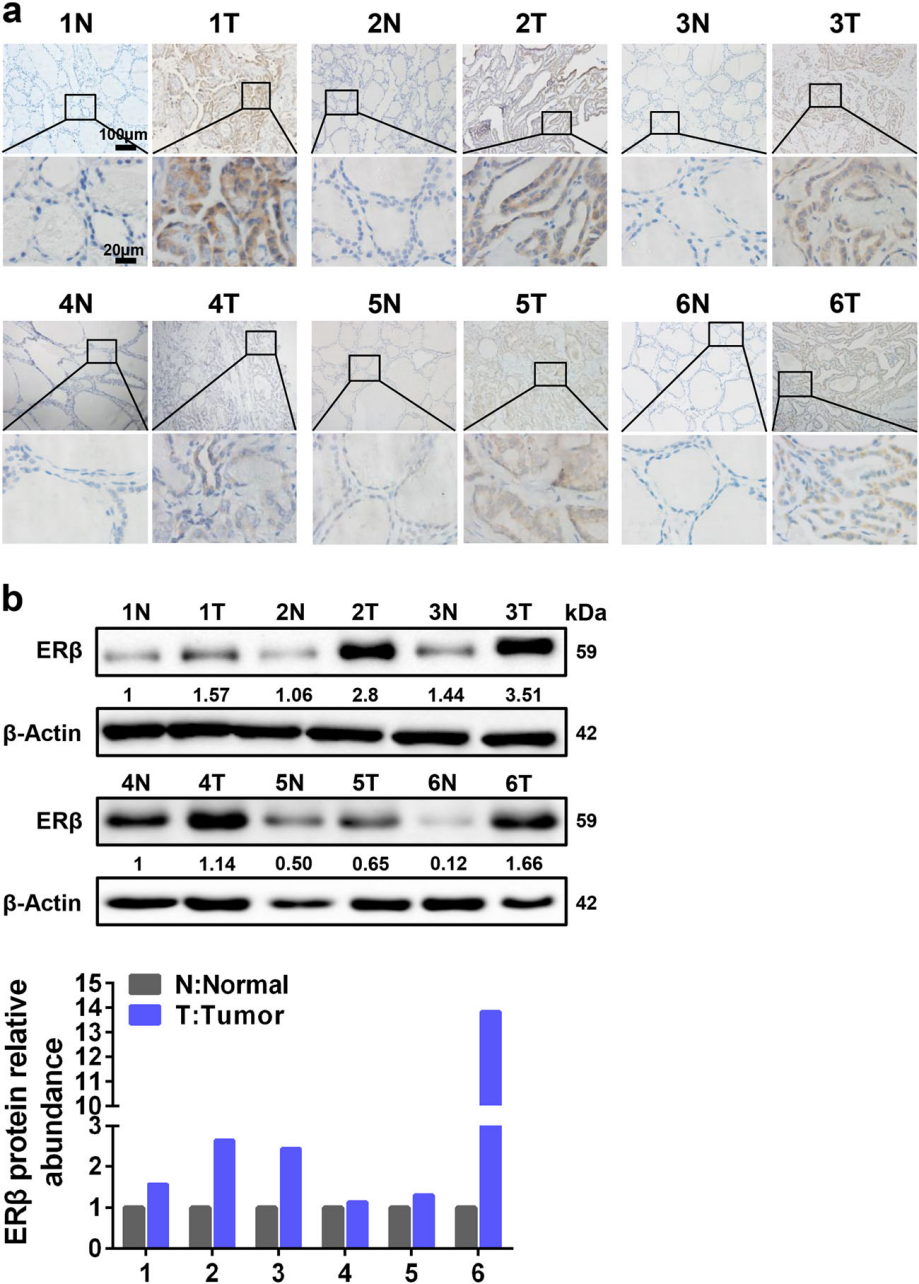
ERβ expression is highly expressed in PTC tissue specimens. **a** Six pairs of PTC tissue specimens (T) and adjacent normal tissue specimens (N) were subjected to IHC staining for ERβ. Representative images were presented. Scale bar represents 100 or 20 μm. **b** ERβ expression was analyzed by western blotting in another six pairs of PTC tissues (T) and adjacent normal tissues (N) (upper panel). Fold change of ERβ expression was normalized to adjacent normal tissue in each pair (lower panel)

To further examine the ERβ expression in clinical samples, we performed immunohistochemistry (IHC) staining to measure ERβ in PTC tissue specimens and the corresponding adjacent tissues. ERβ exhibited higher expression in tumor tissue specimens compared to the corresponding adjacent tissues (Fig. 5a). Next, we assessed the expression of ERβ using western blotting assay in another six pairs of tumor tissue specimens, and similar ERβ expression patterns were also observed (Fig. 5b). These results demonstrate that ERβ is upregulated in PTC tissue specimens.

### Aspirin suppresses E2-induced cancer stemness through decreasing *H19* and ERβ expression

**Fig. 6:**
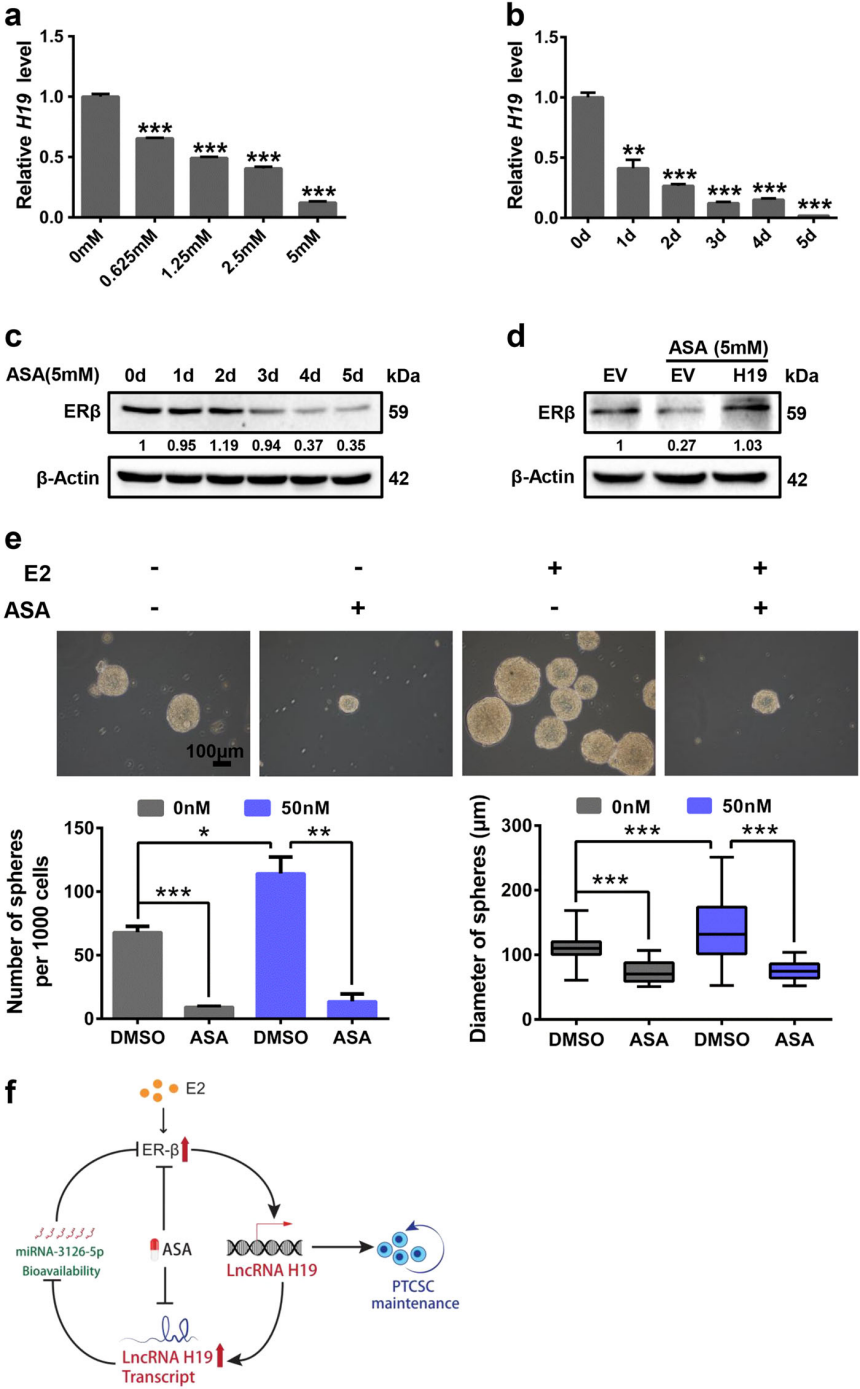
Aspirin suppresses E2-induced cancer stemness through decreasing *H19* and ERβ expression. **a** K-1 cells were treated with aspirin for 3 days. The mRNA expression of *H19* was detected by RT-qPCR in different doses (0, 0.625, 1.25, 2.5, and 5 mM) treatment. The relative *H19* level was normalized to *ACTB*. Data were shown as means ± SD (*n* = 3, ***P < 0.001). **b** K-1 cells were treated with aspirin (5 mM). The mRNA level of *H19* was detected by RT-qPCR at different time points (0, 1, 2, 3, 4, and 5 days). The relative *H19* level was normalized to *ACTB*. Data were shown as means ± SD (*n* = 3, **P < 0.01 and ***P < 0.001). **c** K-1 cells were treated with 5 mM aspirin. The Expression of ERβ was detected by western blotting in different time points (0, 1, 2, 3, 4, and 5 days) treatment. β-Actin acted as the loading control. **d** K-1 cells were transfected with *H19*-overexpressing vector (*H19*) or empty vector (EV), ERβ protein level was detected by western blotting in the absence or presence of ASA for 3 days. β-Actin acted as the loading control. **e** K-1 cells were treated with E2, aspirin and combination E2 with aspirin, sphere forming ability was analyzed. Representative images were presented, the scale bar represents 100 μm. The numbers and size of spheres were counted after culture for 10 days. Data were shown as means ± SD. (*n* = 3, *P < 0.05, **P < 0.01 and ***P < 0.001). **f** Model for the E2-ERβ-*H19* underlies stem-like traits in papillary thyroid carcinoma

Previous studies have demonstrated that aspirin (ASA) possesses antineoplastic actions against a wide range of solid tumors. Upon ASA treatment, *H19* expression was dramatically decreased in both dose-dependent (Fig. 6a) and time-dependent (Fig. 6b) manners. ASA also resulted in a decrease in the protein expression level of ERβ in a time-dependent manner (Fig. 6c). Moreover, *H19*-overexpressing plasmid in parallel with empty vector (EV) was transiently transfected into K-1 cells under ASA treatment. The expression of ERβ was rescued by overexpression of *H19* in the presence of ASA (Fig. 6d and Supplementary Figure 4a). Notably, E2-enhanced sphere formation abilities were substantially attenuated by ASA in K-1 cells (Fig. 6e). In conclusion, these results reveal that *H19* mediates E2-induced stem-like properties through upregulating ERβ expression in PTC cells, which can be inhibited by ASA (Fig. 6f).

## Discussion

In this study, we demonstrate that the induction of ERβ expression by *H19* is critical for PTCSC maintenance. In agreement, ERβ is highly expressed in PTCSCs and promotes PTC stem-like properties (Fig. 1). Screening estrogen-responsive lncRNAs in spheroid cells, we observe that *H19* is significantly elevated in PTCSCs and PTC tissue specimens (Fig. 2). *H19* transcription can be activated by ERβ under E2 treatment, and ablation of *H19* reverses E2-induced stem-like traits in PTC cells (Fig. 3). Moreover, *H19* sponges miR-3126-5p to release ERβ expression, and silencing of ERβ remarkably inhibits E2/*H19*-induced stem-like properties in PTC cells (Fig. 4). In concordance, ERβ also displays a higher expression level in PTC tissue specimens (Fig. 5). Notably, aspirin can antagonize E2-induced stem-like properties through suppressing *H19* and thereby ERβ expression (Fig. 6).

Accumulating studies have revealed correlations between thyroid cancer incidence and ovulatory cycles, pregnancy, and lactation suppressant^28,29^, which suggest a pivotal role of sex hormones, in particular estrogen, in PTC progression. For example, E2 has been shown to stimulate thyroid cancer cell proliferation through increasing the anti-apoptotic protein BCL-2 and decreasing the pro-apoptotic BAX in an ERK1/2-dependent manner^30^. E2 also promotes adhesion, migration, and invasion capabilities via β-catenin in thyroid cancer cells^31^. Recent studies have reported that estrogens are involved in elevating hematopoietic stem-cell self-renewal capabilities in female subjects and more specifically during pregnancy^32^. Although E2 promotes sphere formation abilities, elevates tumorigenicity of PTCSCs and decreases the expression of the differentiation markers in thyroid progenitor cells^9,19^. The detailed mechanisms in which estrogen modulates PTCSCs are still unknown. Recent study reported that *H19* is downregulated in PTC tissues and PTC cell lines^33^. In our study, only clinical specimens of reproductive age were selected as candidates, which were considered to have high estrogen levels. We found that *H19* is upregulated in PTC tissues. In addition, *H19* was elevated in PTCSCs enriched by sphere formation which indicates *H19* plays an important role in PTC stemness. Conversely, silencing of *H19* significantly reverses E2-induced PTC stem-like properties (Fig. 3). *H19* has been reported to be upregulated by E2 via the estrogen–ERα–*H19* signaling axis in breast tumors^20,34^. Furthermore, CLIM interacting with ERα binds to *H19* locus and promotes *H19* expression, which negatively regulates corneal epithelial proliferation^35^. However, the detail mechanism on how ER regulates *H19* expression remains unclear. Our results show that E2 treatment increases lncRNA *H19* transcription via ERβ in PTC cells. Thus, our data contribute to the understanding of the mechanism by which hormones effects on thyroid pathogenesis.

Recent studies have revealed that ERs play critical roles in the PTC development. ERα expression is usually increased in thyroid tumors, while ERβ expression is reduced when compared with normal parenchyma^36,37^. Estrogen-activated ERα mediates the stimulatory effects on PTC proliferation and migration, whereas ERβ has inhibitory actions^8,15–17^. In general, ERα promotes proliferation with an anti-apoptosis effect, while ERβ is related to apoptosis and growth inhibition. For this reason, the ERα/ERβ ratio is helpful in elucidating the thyroid cancer pathophysiology^6,7^. However, ERβ expression was elevated in advanced prostate tumor tissues, which was associated with poor prognosis of hormone-naive patients^38^. Depletion of ERβ attenuated mammosphere formation ability in breast cancer cells and patient-derived breast cancer cells^39^. The fact that these two ERs have distinct distributions in the body and cell subpopulation indicates the different roles of ERα or ERβ maybe cancer-type and cell subpopulation-dependent^40^. Our study firstly reveals that ERβ is highly expressed in PTCSCs and contributes to PTCSC maintenance (Fig. 1). ERβ depletion remarkably reverses *H19-*mediated PTC stem-like capability upon E2 treatment (Fig. 4). Our previous study has shown that *H19* functions as a competitive endogenous RNA (ceRNA) to sponge miRNA let-7, leading to the upregulation of HIF-1α protein expression^41^. Here, we demonstrate that E2-induced *H19* acting as a ceRNA sponge miR-3126-5p to release ERβ expression. Furthermore, whether ERβ, as a key transcriptional factor, transactivates self-renewal genes to maintain PTCSCs requires further exploration.

As ERβ has a critical role in regulating PTCSC maintenance, targeting ERβ could provide a novel therapeutic avenue for advanced PTC patients. A specific ERβ antagonist, 4-[2-phenyl-5,7-bis(trifluoromethyl)pyrazolo[1,5-a]pyrimidin-3-yl]phenol (PHTPP), has been shown to be effective in many cancer types. For examples, bladder cancer burden and mortality can be controlled by PHTPP treatment in the carcinogen-induced bladder cancer models^42^. Consistently, PHTPP can also attenuate 27-hydroxycholesterol-induced cell proliferation in prostate cancer cells^43^. However, the clinical application of PHTPP has been limited by its high toxicities and inferior selectivity^39^. In particular, a recent study has reported that treatment with PHTPP even promotes prostate cancer invasion^44^. As a result, new medications are urgently needed to replace PHTPP for targeting ERβ-induced CSCs. Accumulative evidence has demonstrated that the FDA-proved anti-inflammatory drug aspirin (ASA) can exert inhibitory effects on CSCs. For example, aspirin can restrict cancer stem-like properties by decreasing the expression of stemness-related factors in pancreatic cancer and has no significant toxic effects on normal cells^45^. In addition, a previous study has also shown that aspirin inhibits breast cancer stem cell properties via targeting NF-κB signaling^46^. Notably, our findings were the first to demonstrate that aspirin markedly inhibits PTC stemness through decreasing both lncRNA *H19* and ERβ expression (Fig. 6).

In summary, our studies reveal that ERβ–*H19* positive feedback loop promotes PTC stem-like traits under E2 treatment. In addition, this novel PTCSC regulatory mechanism could be inhibited by the clinically approved medicine aspirin, thus providing a potential therapeutic opportunity for aggressive PTC.

## Materials and methods

### Clinical samples

Following informed consent from patients and approved by the Institutional Ethics Review Board of first Affiliated Hospital of Dalian Medical University, all PTC samples and PTC paraffin tissue specimens used in this study were obtained from the first Affiliated Hospital of Dalian Medical University. Samples were frozen in liquid nitrogen immediately after surgical resection for later mRNA and protein extraction.

### Cell lines

The human thyroid cancer cell lines (TPC-1 and K-1) and 293T cells were purchased from American Type Culture Collection (ATCC, Manassas, VA, USA). The cell lines were authenticated at ATCC before purchase by their standard short tandem repeat DNA typing methodology. Each cell line was cultured in its standard medium as recommended by ATCC. TPC-1 cells, K-1 cells, and 293T cells were maintained in Dulbecco’s modified Eagle’s medium (Invitrogen, Carlsbad, CA, USA) supplemented with 10% (v/v) FBS. All cells were incubated at 37 °C in a humidified incubator containing 5% CO_2_.

### Chemicals and E2 treatment

All Chemicals including 17β-estradiol (E2), charcoal, and aspirin were obtained from Sigma (St. Louis, MO, USA). E2 was dissolved in ethanol and aspirin was dissolved in DMSO following manufacturer’s instructions. Before E2 treatment, cells were cultured in phenol red-free DMEM (Invitrogen, Carlsbad, CA, USA) medium supplemented with 10% charcoal-stripped FBS for three generations. Subsequently, we treated the cells with various concentrations of E2 as indicated and ethanol as vehicle control.

### Sphere formation assay

Sphere formation assay was conducted in serum-free DMEM/F12 (Gibco, Carlsbad, CA, USA) supplemented with 2% (v/v) B27 (Invitrogen, Carlsbad, CA, USA), 20 ng/ml EGF (Sigma, St. Louis, MO, USA) and 20 ng/ml basic FGF (BD Biosciences, CA, USA). Dissociated single cells (600 or 1000) were seeded into 2 mL medium and propagated in six-well ultra-low attachment plates (Corning, NY, USA) and subsequently cultured at 37 °C in 5% CO_2_. Triplicate wells were set up. Sphere numbers were quantified at day 10 or 14. The spheres greater than 50 μm diameter were counted at 10× or 20× magnification under Olympus microscope.

### RNA extraction and RT-qPCR analysis

Total RNA was extracted using Trizol reagent (Invitrogen, Carlsbad, CA, USA) following manufacturer’s instructions, which was used to generate cDNA by using EasyScript One-Step gDNA Removal and cDNA Synthesis SuperMix Kit (TransGene Biotech, Beijing, China) with a random primer. RT-qPCR was performed using specific SYBR Select Master Mix (Invitrogen, Carlsbad, CA, USA) as recommended by the manufacturer. The relative mRNA levels were normalized to *ACTB*. The primers used were listed in Supplementary Table 1.

### Western blotting

Cells were washed with ice-cold PBS and lysed in RIPA lysis buffer with freshly added cocktail protease inhibitor (Thermo Scientific, Rockford, IL, USA) on ice. Equal amounts of proteins were separated by SDS-PAGE and transferred to nitrocellulose membranes (Millipore, County Cork, Ireland). The membranes were blocked with 5% fat-free milk in TBST at room temperature for 60 min and then incubated with indicated primary antibodies followed by incubation with peroxidase-conjugated secondary antibodies (Thermo Scientific, Rockford, IL, USA) at room temperature for 60 min. The protein bands were detected and analyzed with an enhanced chemiluminescence kit (Amersham, Marlborough, MA, UK) using Bio-Rad ChemiDoc XRS^+^ Imaging System according to the manufacturer’s instructions. The Primary antibodies were used as follows: mouse anti-β-Actin (Proteintech, Wuhan, China), rabbit anti-ERβ (Bioworld Technology, Louis Park, MN, USA), rabbit anti-OCT4 (Cell Signaling Technology, Danvers, MA, USA), rabbit anti-NANOG (Cell Signaling Technology, Danvers, MA, USA).

### IHC staining and scoring

For this trial, PTC paraffin tissues were sectioned into 5 μm slices. SPlink Detection Kits (Biotin-Streptavidin HRP Detection Systems, ZSGB-BIO, Beijing, China) and a DAB Kit (ZSGB-BIO, Beijing, China) were used. Briefly, xylene and gradient ethanol were used for dewaxing and rehydration respectively. To block endogenous peroxidase activity, slides were immersed in 3% hydrogen peroxide for 10 min. For epitope retrieval, slides were microwave treated in indicated target retrieval solution for 5 min, three times. After blocking, the slides were incubated with the primary antibody of ERβ (Abcam, Cambridge, MA, USA), NANOG (Abcam, Cambridge, MA, USA), overnight at 4 °C, respectively. Then the slides were incubated with the HRP-labeled anti-rabbit IgG secondary antibody for 30 min and HRP for 20 min at room temperature respectively. Subsequently, DAB was used to stain the slides. Finally, the slides were counterstained with hematoxylin, dehydrated with gradient ethanol. Images were taken at 20× and 100× magnification by Olympus microscope. The immunostaining was observed and scored by two independent experienced pathologists using light microscopy (magnification 20×). The intensity of staining and the proportion of positive cells were used to evaluate the immunostaining. The staining intensity was graded as follows: absent staining as “0”, weak staining as “1”, intermediate staining as “2”, and strong staining as “3”. The percentage of positive cells score was ranked from 0 to 100%. Multiplying the percentage of positive cells score and the intensity score as the final score for each case. For ERβ, 67 was the median level of the final scores of all cases. For NANOG, 57 was the median level of the final scores of all cases. Stained tissues with a final score <median level was further classified as low, whereas tissues with a final score ≥median level were determined as high.

### ALDH staining

For ALDH staining, the ALDH^+^ population was detected by ALDEFLUOR kit (Shanghai Stem Cell Technology Co. Ltd, Shanghai, China) following the manufacturer’s instructions. In brief, K-1 or TPC-1 (siNC and siERβ) cells (1 × 10^6^/mL) were analyzed on a BD C6 flow cytometer (USA) after staining in ALDH1 substrate containing assay buffer for 30 min at 37 °C in dark. The negative control was treated with diethylaminobenzaldehyde (DEAB), a specific ALDH inhibitor.

### Xenograft assay

K-1 (NTC and shERβ) cells (1 × 10^6^) were subcutaneously injected into BALB/c nude female mice (4–6 weeks old, *n* = 5). The tumor volumes were measured by calipers once every three days, estimated using the formula = 0.5 × *a* × *b*^2^ (*a* and *b* were the long and short diameter of the tumors respectively). After 23 days, the mice were sacrificed, and the tumor xenografts immediately dissected.

### Fluorescent in situ hybridization

A fragment of *H19* designed as its probe was used and labeled with digoxigenin (DIG)-UTP (Roche, Mannheim, Germany) using the mMESSAGE T7 Ultra In Vitro Transcription kit (Ambion, Austin, TX, USA) in accordance with the manufacturer’s directions. Slides were hybridized with probes overnight, washed twice with 50% formamide/2 × saline sodium citrate (SSC) and twice with 2 × SSC at 50 °C for 5 min each time, then incubated with 1:500 diluted sheep anti-Dig (lnvitrogen, Carlsbad, CA, USA) for 1 h at room temperature, followed by counterstained with DAPI (1 μg/ml), visualized using a confocal microscope (Leica, Wetzlar, Germany). Probe sequences were listed in Supplementary Table 1.

### Lentivirus infection and transient transfection

Lentiviral-mediated short hairpin RNA (shRNA) directed against *H19* and ERβ were purchased from GenePharma, Suzhou, China. For shRNA lentiviruses infection, cells were infected in 6-cm dishes and subsequently split into 10-cm dishes in the presence of 2 μg/ml puromycin (Sigma, St. Louis, MO, USA) for selection over 72 h. The cells stably expressing shH19 or shERβ were chosen, respectively. shRNA sequence used were listed in Supplementary Table 1. Transient transfection was performed by using Lipofectamine 3000 (Invitrogen, Carlsbad, CA, USA) according to the manufacturers’ protocols.

### Plasmids

Promoters of *H19* (−788/+44), *H19* (−502/+44) were amplified from 293T genomic DNA and inserted into pGL3-Basic (Clontech, CA, USA) to generate the pGL3-H19-WT (−788/+44) and pGL3-H19-Del (−502/+44), respectively. The E2 responsive element (ERE) was mutated (pGL3-H19-Mut) by site-directed mutagenesis using PCR. Sequence of ESR2 3′UTR was amplified from 293T cDNA and inserted into psiCHECK2 vector to generate psi-ESR2-WT. The miRNA response element (MRE) of miR-3126-5p in ESR2 3′UTR region was mutated (psi-ESR2-Mut) by site-directed mutagenesis using PCR. *H19*-expressing plasmids were constructed as previously described^47^. The miR-3126-5p binding sequence of *H19* mutation (H19-Mut) was generated by site-directed mutagenesis using PCR. pRL-SV40 was purchased from (Clontech, CA, USA). All the primers used in plasmid construction were listed in Supplementary Table 1.

### siRNAs, microRNA mimics, and microRNA inhibitors

siRNAs specifically targeting ERβ, siRNA control, miR-127-5p, miR-876-3p, miR-1976, miR-3126-5p, miR-3198, miR-4268 mimics and negative control, miR-3126-5p inhibitor and negative control were all purchased from GenePharma, Suzhou, China. All sequences were listed in Supplementary Table 1.

### Dual-luciferase reporter assays

Luciferase activity was measured using the Dual-Luciferase Reporter Assay system (Promega, Madison, WI, USA) according to the manufacturer’s instructions. Growth media were removed, and cells were washed with cold PBS. Passive lysis buffer (200 μL per well) was added with gentle rocking for 15 min at room temperature. Lysates (50 μL) were transferred in black 96-well plate (Corning, NY, USA). Firefly and Renilla luciferase activity were assayed sequentially to the cell lysate in each well. For each luminescence reading, there would be a 2 s pre-measurement delay after injector dispensing assay reagents into each well, followed by a 10 s measurement time. For pGL3 reporter system, transcriptional activity was calculated as the ratio of firefly luciferase activity (reporter) to Renilla luciferase activity (control). For psiCHECK2 reporter system, the RNA stability was calculated as the ratio of Renilla luciferase activity (reporter) to firefly luciferase activity (control). Results represented the average of triplicate samples from three independent experiments.

### Statistical analysis

Data were expressed as means ± SD of three independent experiments with GraphPad Prism software. The Student’s *t*-test was used to make a statistical comparison between groups. Pearson’s correlation test was used to examine the correlation between ERβ and NANOG by IHC staining. Statistical Package for Social Sciences (SPSS) software (version 24.0) was used for Statistical analysis in this study. *P < 0.05, **P < 0.01 and ***P < 0.001 were considered statistically significant.

## Electronic supplementary material

Supplementary Figure legends

Supplementary Table 1

Supplementary Figure 1

Supplementary Figure 2

Supplementary Figure 3

Supplementary Figure 4
