# Supplementary material for: Estrogen receptor β upregulated by lncRNA-H19 to promote cancer stem-like properties in papillary thyroid carcinoma
Source: Cell Death Dis. 2018 Nov 2;9(11):1120. doi: 10.1038/s41419-018-1077-9 (PMC6214949; doi:10.1038/s41419-018-1077-9)
Supplement: Supplementary file 1 — Supplementary Figure legends [file 41419_2018_1077_MOESM1_ESM.docx]

**Supplementary Figure Legends**

***Supplementary Figure 1 (related to Figure 1)***

(a) *Tg* and *TSHR* mRNA expression in the spheroid cells and monolayer cells of TPC-1 cells and K-1 cells were analyzed by RT-qPCR. Data were shown as means ± SD (n=3, *P<0.05 and ***P<0.001). (b) The protein levels of ERβ and stemness-related factors OCT4 and NANOG were measured in siNC and siERβ TPC-1 cells. β-Actin was included as the loading control. (c) The proportions of ALDH^+^ cells were compared between siNC and siERβ in TPC-1 cells. (d) The interfering efficiencies of the lentivirus ERβ-targeting shRNAs (shERβ-1 and shERβ-2) were confirmed by RT-qPCR and compared with non-targeting control (NTC) in K-1 cells. Relative mRNA levels were normalized to *ACTB*. Data were shown as means ± SD (n=3, **P<0.01). (e) The interfering efficiency of the lentivirus ERβ-targeting shRNAs (shERβ-1 and shERβ-2) were confirmed by RT-qPCR, compared with non-targeting control (NTC) in TPC-1 cells. Relative mRNA levels were normalized to *ACTB*. Data were shown as means ± SD (n=3, ***P<0.001). (f) Sphere formation assays were performed in ERβ depletion TPC-1 cells. Representative images were presented (up), the scale bar represents 100 μm. The numbers and size of spheres were counted after culture for 10 days (down). Data were shown as means ± SD (n=3, *P<0.05 and ***P<0.001).

***Supplementary Figure 2 (related to Figure 3)***

(a-b) Sphere formation was performed under 50 nM E2 treatment in TPC-1 cells and K-1 cells. Representative images were presented (left), the scale bar represents 100 μm. The numbers and size of spheres were counted after culture for 14 days (middle and right). Data were shown as means ± SD (n=3, *P<0.05 and ***P<0.001). (c) The mRNA level of a panel of core pluripotency factors (*NANOG*, *SOX2* and *POU5F1*) were confirmed by RT-qPCR at the presence or absence of E2 in TPC-1 spheroid cells and K-1 spheroid cells. The relative mRNA levels were normalized to *ACTB*. Data were shown as means ± SD (n=3, *P<0.05, **P<0.01 and ***P<0.001, respectively). (d) TPC-1 spheroid cells and K-1 spheroid cells were treated with E2 (50 nM) for 36 hours. The mRNA level of *ESR1* and *ESR2* were measured by RT-qPCR. The relative mRNA levels were normalized to *ACTB*. Data were shown as means ± SD (n=3, ***P<0.001). (e) *H19* promoter sequence (-788 to +44) with E2 responsive element (ERE) or truncated sequence (-502 to +44) without ERE, was cloned into the pGL3 luciferase reporter. (f) The silencing efficiency of siERβ was detected by RT-qPCR in K-1 cells. The relative *ESR2* levels were normalized to *ACTB*. Data were shown as means ± SD (n=3, ***P<0.001). (g) The interfering efficiency of the lentivirus *H19*-targeting shRNAs in TPC-1 cells and K-1 cells were confirmed by RT-qPCR, compared with non-targeting control (NTC). The relative *H19* levels were normalized to *ACTB*. Data were shown as means ± SD (n=3, ***P<0.001). (h) Sphere formation abilities of TPC-1 under different conditions were compared. Representative images were presented, the scale bar represents 100 μm. The numbers and size of spheres were counted after culture for 10 days. Data were shown as means ± SD. (n=3, *P<0.05, **P<0.01 and ***P<0.001).

***Supplementary Figure 3 (related to Figure 4)***

(a) *ESR2* mRNA expression was analyzed by RT-qPCR in shH19 K-1 cells and non-targeting control (NTC) cells. The relative *ESR2* mRNA level was normalized to *ACTB*. Data were shown as means ± SD (n=3, ***P<0.001). (b) K-1 (shH19-3) cells and NTC cells were treated with or without E2 for 36 hours. ERβ expression was analyzed by western blotting. β-Actin acted as the loading control. (c) *ESR2* 3’UTR putative binding miRNAs (red area) and *H19* putative binding miRNAs (blue area) were predicted using bioinformatic tool ‘miRanda’ (http://www.microrna.org). Six candidate miRNAs presented in both categories were selected for further analysis. (d) ERβ protein expression was detected in K-1 cells transfected with six candidate miRNA mimics compared with NC. β-Actin was used as the loading control. (e) ERβ protein expression was detected in K-1 cells transfected with miR-3126-5p inhibitor compared with NC. (f) Putative miR-3126-5p miRNA response element (MRE) on *ESR2* 3’UTR sequence predicted online was shown (upper panel); MRE sequence was cloned into psiCHECK2 vector as luciferase reporter (lower). (g) NTC and shERβ-1 K-1 cells were transfected with *H19*-overexpressing vector (*H19*) or empty vector (EV), the RNA level of *H19* was quantified by RT-qPCR 60 hours post transfection. The relative *H19* levels were normalized to *ACTB*. Data were shown as means ± SD (n=3, **P<0.01 and ***P<0.001).

***Supplementary Figure 4 (related to Figure 6)***

(a) K-1 cells were transfected with *H19*-overexpressing vector (*H19*) or empty vector (EV), *H19* RNA level was detected by RT-qPCR in the absence or presence of ASA for 3 days. The relative *H19* levels were normalized to *ACTB*. Data were shown as means ± SD (n=3, ***P<0.001).
