# Supplementary material for: Estrogen receptor β upregulated by lncRNA-H19 to promote cancer stem-like properties in papillary thyroid carcinoma
Source: Cell Death Dis. 2018 Nov 2;9(11):1120. doi: 10.1038/s41419-018-1077-9 (PMC6214949; doi:10.1038/s41419-018-1077-9)
Supplement: Supplementary file 2 — Supplementary Table 1 [file 41419_2018_1077_MOESM2_ESM.pdf]

The primers used in RT-qPCR are shown

Genes	Forward Primer 5′ >3′	Reverse Primer 5′ >3′
UCA1	CTCTCCATTGGGTTCACCATTC	GCGGCAGGTCTTAAGAGATGAG
H19	TACAACCACTGCACTACCTG	TGGCCATGAAGATGGAGTCG
TC1500845	ACCACGACTCCCAAGAGGTA	CAGCTGCGATGGTGAGAACT
TC0101441	CAAGGCAGGTGAGAACGAGT	CTCGACTTAGGGAGCTGCAC
ROR	CCAGGACAATGAAACCAC	TGGAGCAGGTATGAGATT
MALAT1	GGATCCTAGACCAGCATGCC	AAAGGTTACCATAAGTAAGTTCCAGAAAA
NEAT1	CCAGTTTTCCGAGAACCAAA	ATGCTGATCTGCTGCGTATG
SRA1	TGATGACATCAGCCGACGCCT	GCTGCAGATTTCTCTTCATTG
HOTAIR	CAGTGGGGAACTCTGACTCG	GTGCCTGGTGCTCTCTTACC
BC200	AGACCTGCCTGGGCAATATAGC	GTTGTTGCTTTGAGGGAAGTTACG
RP11-445H22.4	GTAAAGCCATCACCAGGACAACC	CTCCCTAACAGAAGCCCACCA
TC01000223	ATGAGGGCTCTGCTCTATGAATGG	GGCTTGTTCAGTGTCTGTTAAGGGT
TC01001686	GGCTACTTACATGGTCCAGCA	TAGCATGGAAAGGACCACTGC
ESR1	CCACCAACCAGTGCACCATT	GGTCTTTTCGTATCCCACCTTTC
ESR2	GTCACTAACTTGGAAGGTGGG	AGATGTGATAACTGGCGATGG
NANOG	ACCTATGCCTGTGATTTGTGG	AGTGGGTTGTTTGCCTTTGG
SOX2	TCAGGAGTTGTCAAGGCAGAG	CGCCGCCGATGATTGTTATTA

POU5F1	GTGGAGGAAGCTGACAACAA	GCCGGTTACAGAACCACACT
TSHR	TGGTGTGGTTCGTTAGTCTG	AGTTTGTAGTGGCTGGTGAG
Tg	TCGTCTTGCCCTTCAGTTTAC	GTTAAATGTGCCTCTTGTGCC
pre-H19	CCCAGAACCCACAACATGAA	GAGCTGGGTAGCACCATTT
**ACTB**	TTGCCGACAGGATGCAGAAGGA	AGGTGGACAGCGAGGCCAGGAT

shRNA sequences

**Name**	**5′ >3′**
**shERβ-1**	CTCACCATCTAGCCTTAATTC
**shERβ-2**	GGTCGTGTGAAGGATGTAAGG
**shH19-1**	GAGTTAGCAAAGGTGACATCT
**shH19-2**	GACGTGACAAGCAGGACATGA
**shH19-3**	GCACTACCTGACTCAGGAATC
**NTC**	TTCTCCGAACGTGTCACGTTTC

RNA probe sequences for H19 in FISH assay

**Name**	**Forward Primer 5′ >3′**	**Reverse Primer 5′ >3′**
***H19* probe**	AGCTAGAGGAACCAGACCTCATCA	AATGGAATGCTTGAAGGCTGC

siRNA sequences

Name	5′>3′
**siERβ-1**	GCCCAAAUGUGUUGUGGCCTT
**siERβ-2**	UCAUUAUGUCCUUGAAUGCTT
**siNC**	UUCUCCGAACGUGUCACGUTT

miRNA mimic

Name	5′>3′
**miR-127-5p**	CUGAAGCUCAGAGGGCUCUGAU
**miR-876-3p**	UGGUGGUUUACAAAGUAAUUCA
**miR-1976**	CCUCCUGCCCUCCUUGCUGU
**miR-3126-5p**	UGAGGGACAGAUGCCAGAAGCA
**miR-3198**	GUGGAGUCCUGGGGAAUGGAGA
**miR-4268**	GGCUCCUCCUCUCAGGAUGUG
**NC**	UUCUCCGAACGUGUCACGUTT

miRNA inhibitor

Name	5′>3′
**miR--3126-5p**	UGCUUCUGGCAUCUGUCCCUCA
**NC**	CAGUACUUUUGUGUAGUACAA

The primers used in plasmid construction are shown

plasmid	Forward Primer 5′ >3′	Reverse Primer 5′ >3′
**pGL3-H19-WT**	ATATGGTACCACAACCCTCACCAAAGGCCAA	ATATAAGCTTCTCCCTCACCCTGCTCCTCG
**pGL3-H19-Del**	CGGGGTACCGGTCAACTGGATGGGAATCGG	ATATAAGCTTCTCCCTCACCCTGCTCCTCG
**pGL3-H19-Mut**	ACCGGGGTGCCCAGCGGCTGTCCGACCTCTG TCGTGCGGAAACCGCGGTG	GGTCACCGCGGTTTCCGCACGACAGAGGTC GGACAGCCGCTGGGCACCCC
**psiCHECK2-ESR2-WT**	CCGCTCGAGCGCCTGGCCCTGAGGTGAACTG	ATAAGAATGCGGCCGCCCAAATGAGGGAC CACACAGCAGAAAGATG
**psiCHECK2-ESR2-Mut**	GGGCTTCATCTTTCTGCTGTGTGTATTTATAT TTGGGCGGCCGCTGGCCGCAATAA	AGATATTTTATTGCGGCCAGCGGCCGCCCA AATATAAATACACACAGCAGAAAGATG
**pcDNA6b-H19-Mut(miR-3126-5p binding sites)**	GCCGGGCAGGTGAGTCCCTAAAAAACCCAGG CCTCGCTTCCCCAGC	GGGAAGCGAGGCCTGGGTTTTTTAGGGACT CACCTGCCCGGCAGA
