# Supplementary figures and images for: Estrogen receptor β upregulated by lncRNA-H19 to promote cancer stem-like properties in papillary thyroid carcinoma
Source: Cell Death Dis. 2018 Nov 2;9(11):1120. doi: 10.1038/s41419-018-1077-9 (PMC6214949; doi:10.1038/s41419-018-1077-9)

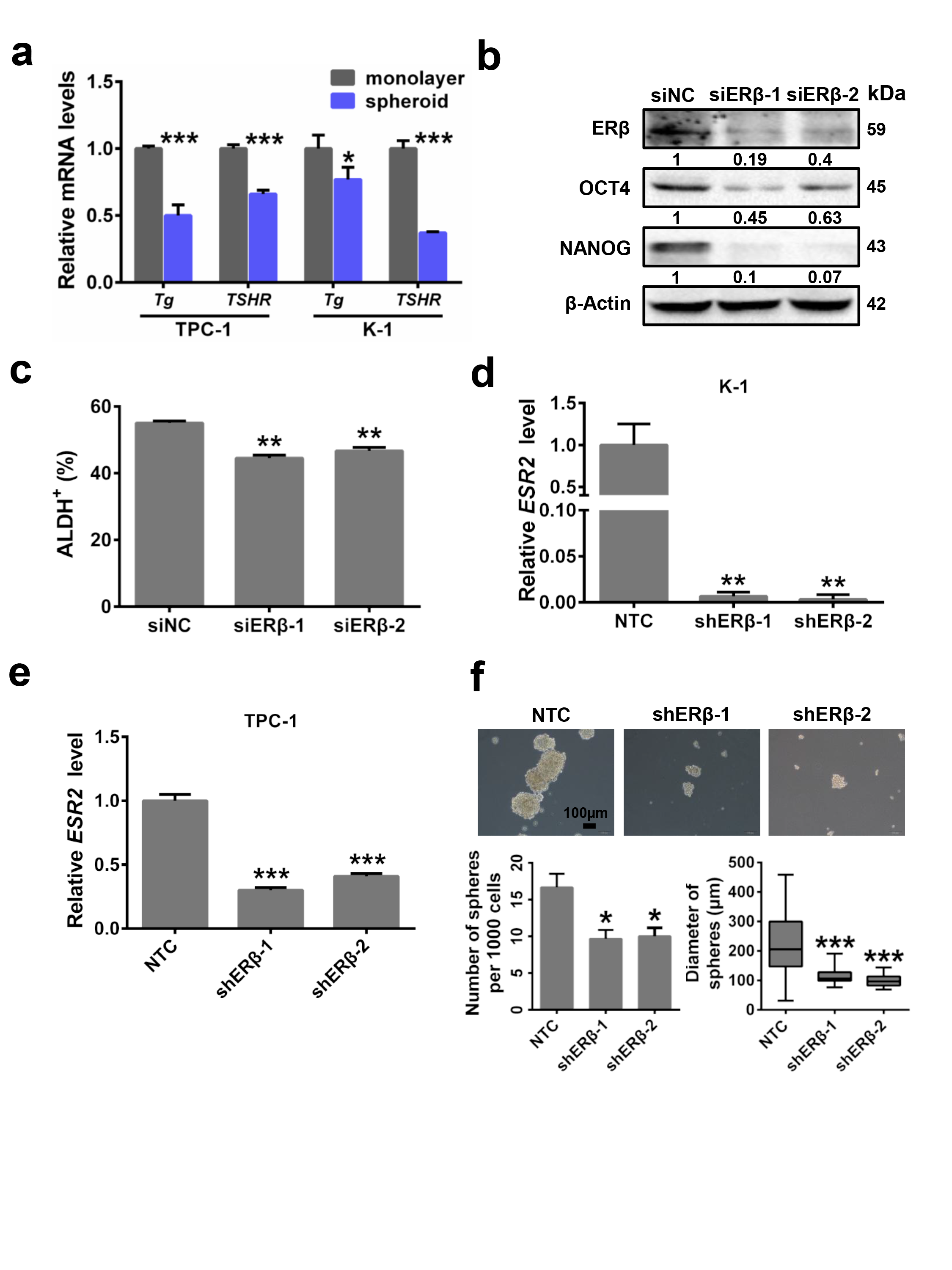

Supplement: Supplementary file 3 — Supplementary Figure 1 [file 41419_2018_1077_MOESM3_ESM.tif]

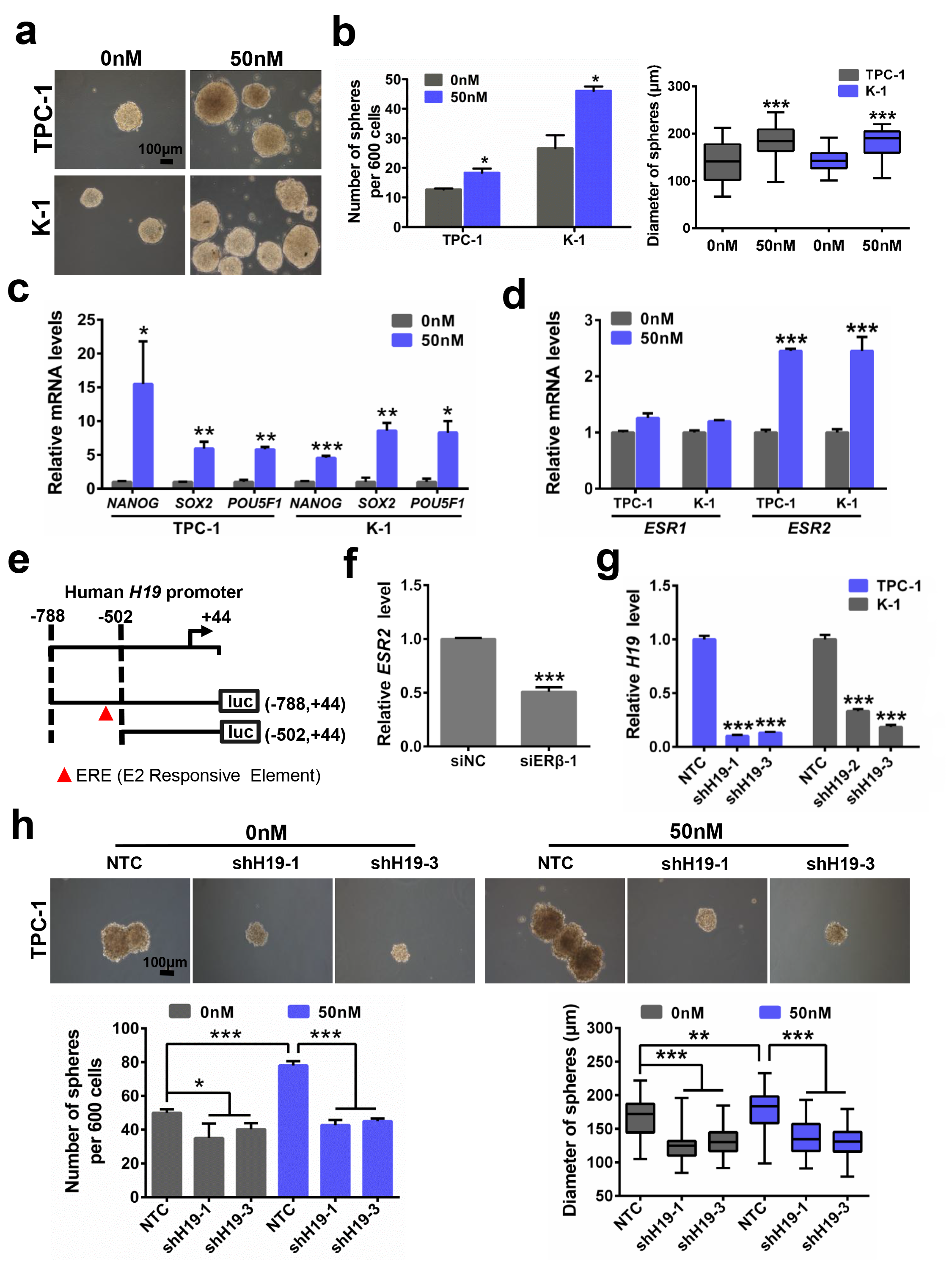

Supplement: Supplementary file 4 — Supplementary Figure 2 [file 41419_2018_1077_MOESM4_ESM.tif]

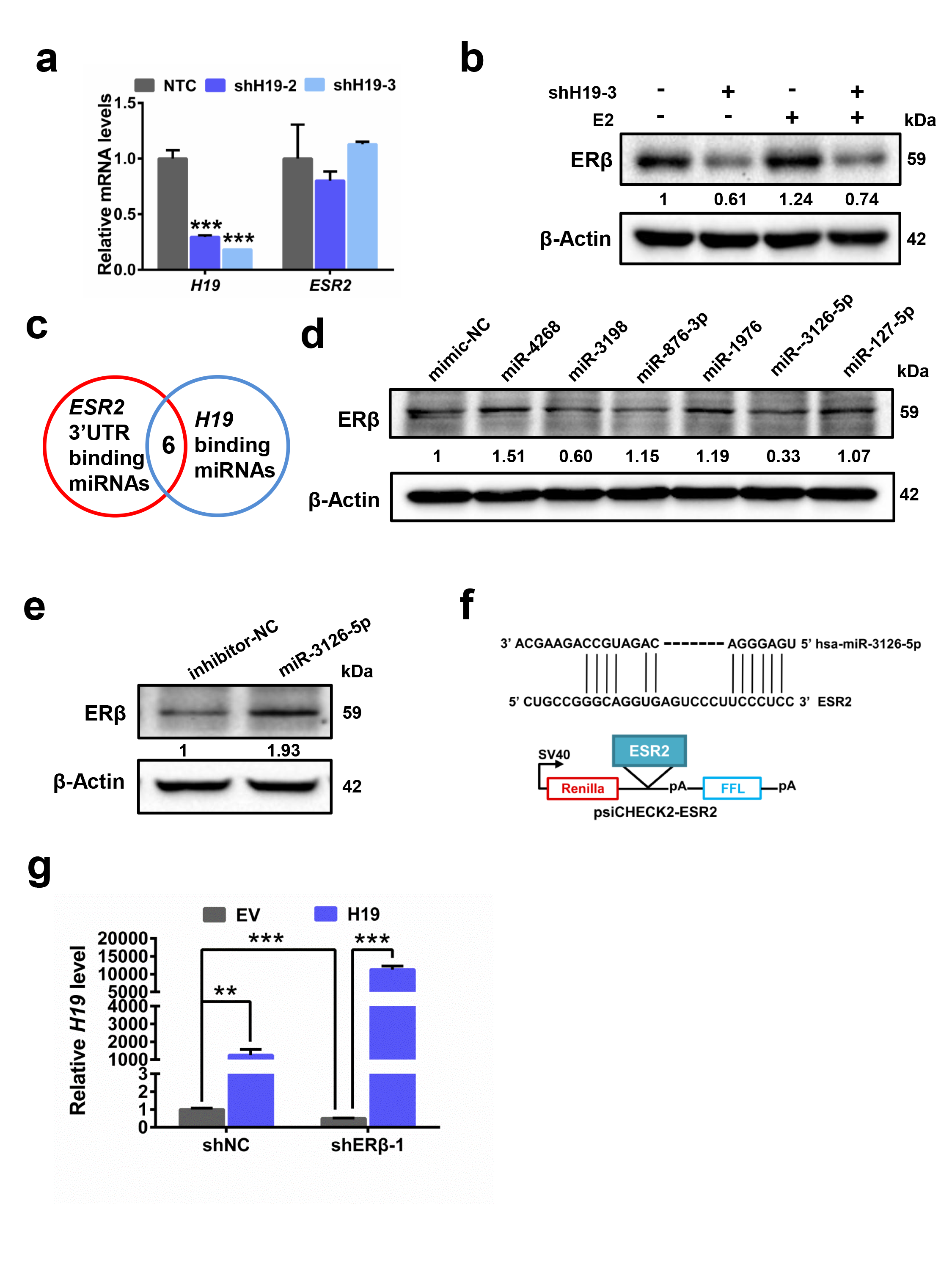

Supplement: Supplementary file 5 — Supplementary Figure 3 [file 41419_2018_1077_MOESM5_ESM.tif]

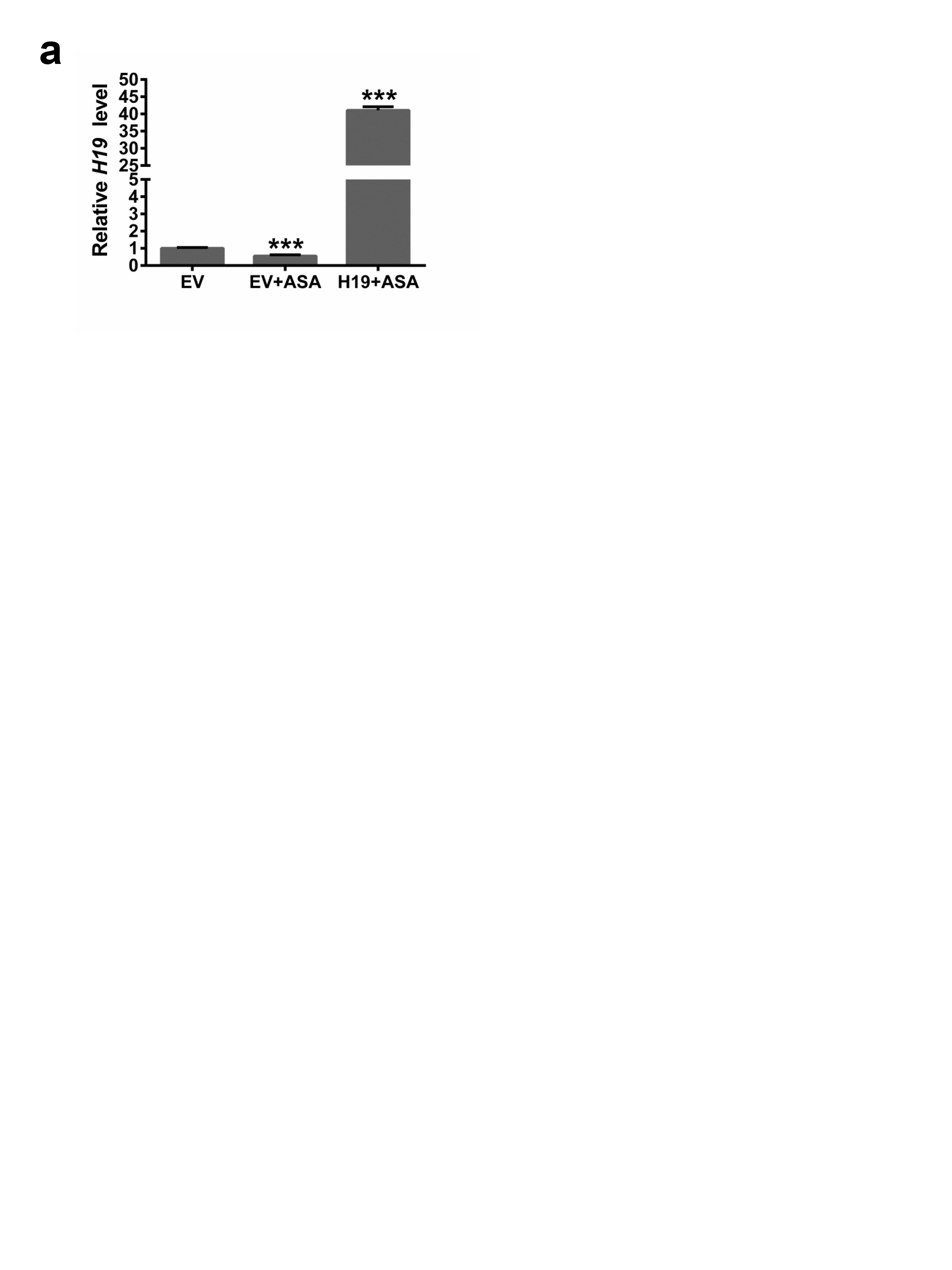

Supplement: Supplementary file 6 — Supplementary Figure 4 [file 41419_2018_1077_MOESM6_ESM.tif]
